# Salicylic Acid Effects on Flue-Cured Tobacco Quality and Curing Characteristics During Harvesting and Curing in Cold-Stressed Fields

**DOI:** 10.3389/fpls.2020.580597

**Published:** 2020-10-30

**Authors:** Xian He, Tianxiang Liu, Ke Ren, Jie Chen, Gaokun Zhao, Binbin Hu, Anchuan Xu, Yan Jin, Yanmei Zhu, Congming Zou

**Affiliations:** ^1^Yunnan Academy of Tobacco Agricultural Sciences, Kunming, China; ^2^Tobacco Monopoly Administration of Yunnan Wenshan Prefecture, Wenshan, China

**Keywords:** abiotic stress, physiology and biochemistry, carbon metabolites, nitrogen metabolites, yield

## Abstract

Salicylic acid (SA) can induce plants to actively enhance abiotic stress resistance. Spraying SA to prevent cold stress in flue-cured tobacco fields can provide theoretical support and practical guidance for the actual protection from cold stress in fields at high altitude in Yunnan. The experiment was performed in Jianchuan County Yunnan Province, China. Honghuadajinyuan, a flue-cured tobacco variety with cold resistance, was used as the research object. SA was tested at two concentrations (0.05 [SA-1] and 0.1 [SA-1] mol L^–1^) relative to an untreated control (Control) to compare the quality of fresh tobacco leaves, curing characteristics, enzyme activity of antioxidants, and quality of the first-cured tobacco leaves. The tissue structure thickness, SPAD, and plastid pigment content of fresh tobacco leaves were least in the control; there was no significant difference between SA-1 and SA-2. The change of moisture content during curing was SA-1 > SA-2 > Control, and the water loss rate was Control > SA-2 > SA-1, and both varied greatly at 38–48°C. In each curing stage, the carbon and nitrogen metabolites and polyphenols changed most rapidly at 38°C, and the sugar metabolites changed as follows: Control > SA-1 > SA-2. The activities of the antioxidant enzymes superoxide dismutase, peroxidase, and catalase in fresh tobacco leaves were SA-1 > SA-2 > Control. Malondialdehyde content and the inactivation rate of antioxidant enzymes during curing was Control > SA-2 > SA-1. The economic character and sensory smoking quality of flue-cured tobacco leaves were SA-1 > SA-2 > Control. In high-altitude tobacco planting areas prone to cold stress in the field, early warning weather forecast and field spraying 0.05 mol L^–1^ SA are beneficial to protect and improve the quality of fresh tobacco leaves, curing characteristics, antioxidant system enzyme activities, and the quality of flue-cured tobacco leaves.

## Introduction

Cold stress occurs at low but above-zero temperatures, and the agricultural loss caused by cold injury in the world costs hundreds of billions of dollars each year. The damage to crops is mainly reflected in the destruction of the cytoplasmic membrane in the plant’s physiological and biochemical systems and the damage to the structure and activity of protective enzymes (Kumar et al., 2010). Cold stress can also inhibit photosynthesis and respiration (Wang et al., 2008; Yadav, 2010). After cold stress, plants will show phenotypic symptoms such as loss of water, wilting of leaves, fading of green, yellowing, decrease in leaf area, and even tissue death (Hussain et al., 2018).

Many warm season crops are extremely sensitive to cold stress. Cold injury in maize (*Zea mays*) from emergence to maturity will lead to a decrease in seed germination rate, hinder the growth of functional leaves, inhibit photosynthesis, slow dry matter accumulation and filling rate, and finally reduce yield and quality (Prasad et al., 1994). When cotton (*Gossypium hirsutum* L.) encounters a short-term low temperature in the later stage of growth, it will experience significant premature aging, with a 30–40% reduction in output and a large-scale outbreak of leaf spot lesions (Zhao et al., 2012). Soybean (*Glycine max*) suffering from low temperature at flowering will have a rapid decrease in the number of pods and seeds per plant, affecting the formation of seed pods, resulting in a decrease in yield and quality (Yang et al., 2004).

Salicylic acid (*o*-hydroxybenzoic acid) is a type of phenolic plant growth regulator that can be synthesized in trace amounts and that has a positive regulatory effect on plant stress resistance (Madan and Levitt, 2014). Many studies have shown that salicylic acid can reduce crop cell membrane permeability, increase proline content to maintain cell water content, reduce or eliminate membrane lipid peroxidation, and effectively enhance the cold resistance of crops (Agarwal et al., 2005; Ghanbari and Sayyari, 2018). Salicylic acid is widely used to improve the stress ability of crops, especially to reduce membrane lipid peroxidation and cell membrane permeability during cold stress and to enhance the activity of protective enzymes to help eliminate the accumulation of reactive oxygen species in plants (Achuo et al., 2004; Farooq et al., 2008; Supapvanich and Promyou, 2013). Salicylic acid treatment in low temperature stress could reduce the damage of leaf tissue structure of papaya (*Carica papaya* L.) seedlings and maintain the integrity of epidermal cell structure (Mandal et al., 2017). Fan et al. (2012) confirmed that applying 1.0 mmol L^–1^ salicylic acid could effectively increase the chlorophyll content of muskmelon (*Cucumis melo* L. cv. *Yujinxiang*) seedlings in low temperature stress. Janda et al. (1999) pretreated maize seedlings in low temperature stress with 0.5 mmol L^–1^ salicylic acid and found that malondialdehyde (MDA) and O_2_^–^ in seedlings decreased, whereas the activities of protective enzymes such as superoxide dismutase (SOD) and peroxidase (POD) significantly increased.

Yunnan province is located at a low latitude and high altitude, and the tobacco (*Nicotiana tabacum* L.) planting area is densely distributed at 1,300–2,000 m above sea level (ASL). In tobacco planting areas with an altitude of more than 2,000 m ASL, fields have a large temperature difference between day and night, and the temperature drop is common. Cold stress has a great influence on the quality of the fresh tobacco leaves, which has a serious effect on the curing of the flue-cured tobacco, resulting in a substantial reduction in tobacco leaf quality (Bolat et al., 2014). Currently, field research of flue-cured tobacco treated with salicylic acid has mainly focused on the physiological and biochemical mechanisms of stress in the seedling stage of flue-cured tobacco exposed to artificial cold injury. Therefore, it is of great interest to explore the efficacy of applying salicylic acid to prevent cold injury stress on leaf quality and curing characteristics of fresh flue-cured tobacco leaves under fields in high-altitude Yunnan tobacco planting areas.

After cold injury of flue-cured tobacco, with the prolongation of cold stress time and the continuous decrease in temperature, the degree of water loss, wilting, shrinkage, and yellowing of flue-cured tobacco leaves increases. On the other hand, cold stress will promote the formation of waxy layer on the surface of flue-cured tobacco leaves, increasing leaf thickness and decreasing tissue ratio. Plasmolysis occurs, and important organelles such as mitochondria deform and disintegrate (Hetherington and Öquist, 2006). The effect of cold stress on the quality of fresh tobacco leaves is also reflected in inhibiting the formation of chloroplasts and reducing the chlorophyll synthesis rate, resulting in decreased photosynthesis of tobacco leaves (Moon et al., 1995; Wang et al., 2006).

The curing characteristics of tobacco leaves inevitably reflect the differences in the quality of fresh tobacco, including two aspects: “ease of curing” and “resistance to flue-curing” (Ke-ai et al., 2006). During curing, the water loss and yellowing of normal tobacco leaves are easily regulated, and a better quality of flue-cured tobacco leaf can be obtained. Cold-injured tobacco leaves lose water with difficulty in the curing process because of the waxy layer and thickened cell wall formed on its surface (Wang et al., 2006). At the same time, because of the decrease in chlorophyll content of cold-injured tobacco leaves, the yellowing rate of tobacco leaves is faster than the rate of water loss, and the two cannot be coordinated, which also brings difficulties to flue-curing. Many enzymatic browning reactions occur easily because of the damage of cell membranes, which leads to the formation of browning tobacco (Chen et al., 2019).

Carbon and nitrogen metabolism are the most basic metabolic process of flue-cured tobacco that has a significant effect on the quality of flue-cured tobacco (Hongbin, 2007). Cold injury inhibits photosynthesis and the activity of carbon and nitrogen metabolic enzymes in tobacco, decreases the content of photosynthates, and then affects the formation and transformation of starch, disaccharide, protein, nicotine, and total nitrogen (Yang et al., 2004). Polyphenols are important secondary metabolites and aroma precursors of tobacco, which play an important role in the growth, development, yield, and quality of tobacco, and are an important factor to measure the quality of tobacco. Some studies have shown that cold stress can promote the synthesis of polyphenols in flue-cured tobacco seedlings (Dagnon and Edreva, 2003; Wang et al., 2008). By enhancing the cold resistance of crops, salicylic acid can reduce or prevent the quality and curing characteristics of fresh tobacco leaves from being affected by cold stress.

In low temperature stress, the antioxidant protective enzymes in flue-cured tobacco can scavenge reactive oxygen species and maintain a low level of reactive oxygen species. Catalase (CAT), POD, and SOD are important antioxidant protective enzymes in tobacco seedlings under low temperature stress (Xue et al., 2009). In normal circumstances, cold injury will increase the activity of antioxidant enzymes for a short time, and then the enzyme activity will decrease. Salicylic acid can improve the stability of cell membrane and induce and enhance the activities of antioxidant enzymes CAT, POD, and SOD to alleviate the cold injury caused by low temperature stress by reducing the MDA content and electrical conductivity of leaves. The appropriate concentration of salicylic acid could enhance resistance to membrane lipid peroxidation of flue-cured tobacco in low temperature stress, thus resisting the harm of low temperature; a concentration of 1.0 mmol L^–1^ appears best (Agarwal et al., 2005). In addition, there is an important relationship between the metabolism of polyphenols and polyphenol oxidase (PPO) is the key enzyme of enzymatic browning reaction in the curing process. Some studies show that low temperature can significantly reduce PPO activity of fresh tobacco leaves (Dechuan, 2006; Bahari et al., 2015). In the curing stage, because of the increase in temperature and humidity, cold injury cell membrane damage very easily allows enzymatic browning reaction to form browning tobacco. The alleviation of salicylic acid on membrane lipid peroxidation and cell membrane permeability under cold stress can effectively prevent the binding of PPO and polyphenols.

When the high-altitude tobacco-growing areas in Yunnan enter the harvesting and curing period, there are large daily temperature differences between day and night, and cliff cooling often occurs. Periodic rainfall accentuates the cold stress, bringing a great threat to local flue-cured tobacco production. There have been a few recent studies on the application of salicylic acid to prevent cold injury stress of flue-cured tobacco during harvesting and curing period in Yunnan. The mechanism of the effect of salicylic acid on the quality, curing characteristics, chemical composition, yield, and quality of flue-cured tobacco leaves is not clear, so it is of important practical significance to explore the mechanisms by which salicylic acid prevents field cold injury stress of flue-cured tobacco during the harvesting and curing period.

## Materials and Methods

### Experimental Materials

The experiment was performed in 2019 at an altitude of 2,565 m ASL in Laojun Mountain Town, Jianchuan country Yunnan Province (E 99°33′, N 26°31′). The tobacco variety Honghuadajinyuan was used for the experiment and was prepared by floating seedling technology. The young seedlings germinated on membranes were transplanted on April 13, with the row and plant spacing being 120 cm × 60 cm. The seedlings were topped on June 10, during which 15 or 16 leaves were left; the lower leaves were picked on July 03, and curing was completed on September 07. Details of soil nutrient levels at the experiment site are as follows: pH 6.47, organic matter content of 56.19 g kg^–1^, total nitrogen of 2.76 g kg^–1^, total phosphorus of 1.11 g kg^–1^, total potassium of 17.64 g kg^–1^, soluble nitrogen of 210.8 mg kg^–1^, available phosphorous of 91.3 mg kg^–1^, and rapidly available potassium of 285.5 mg kg^–1^. Precipitation of the field growth period is as follows: April: 36 mm, May: 6 mm, June: 220 mm, July: 569 mm, and August: 423 mm. A compound fertilizer designed for growing tobacco was used as the base fertilizer, having N: P_2_O_5_: K_2_O = 12: 10: 25, with the application rate of 150 kg ha^–1^, 18 kg ha^–1^ of nitrogen fertilizer was applied, accompanied by composted farmyard manure (15,000 kg ha^–1^). During top dressing, the application rates of compound fertilizer designed for tobacco, nitrogen fertilizer, and accompanying potassium sulfate (51%) were 75, 9, and 30 kg ha^–1^, respectively. The base fertilizers and fertilizers for top dressing were separately applied after transplanting for 15 and 30 days.

### Experiment Design

For the 2012–2016 period during prophase (early May to mid-June), the average temperature that slowly rose leveled off (mid-June to mid-July), with the late period (mid to late of July) showing volatility in average temperature. On the whole, the diurnal range of temperature in the growing period of field was higher in the early stage (from May to mid-June), lower in the middle stage (from mid-June to early August), and higher in the later stage (from mid– August to late August) with severe irregular fluctuations. Therefore, it can be concluded that in the middle and late periods of flue-cured tobacco production (mid and late August) experienced the greatest temperature fluctuations in Jianchuan, Yunnan (Supplementary Figure 1). Tobacco leaves were affected by high and low temperature, and were vulnerable to cold damage stress, which affects the leaf yield quality. According to the experience of local tobacco farmers and the 2019 weather forecast, August was the main period when the local temperature changes were greatest and the field chilling stress occurs frequently. Therefore, the treatments were applied on August 01. The experiment included three treatments (*n* = 3): no salicylic acid (Control); 0.05 mol L^–1^ salicylic acid (SA-1); 0.1 mol L^–1^ salicylic acid (SA-2). Using a randomized block design, each treatment was 12 m long and 8.5 m wide. Salicylic acid was sprayed every 5 days until field chilling injury occurred, and field sampling and tobacco curing characteristics were analyzed.

A TH12R-EX temperature and humidity recorder (Shenzhen Huahanwei Science and Technology Co., Ltd.) were placed in the test field to record daily temperature and humidity changes, while a WH-2310 wireless weather station (Jiaxing Misu Electronic Co., Ltd.) was placed to record daily temperature, precipitation and wind speed.

According to the regular harvest time of the local middle leaves, the tobacco leaves were picked and poled to ensure that the maturity of the tobacco leaves was balanced and consistent, the same quality was on the same pole, and the density was moderate. The tobacco leaves were cured in the local dense curing barn.

**FIGURE 1:**
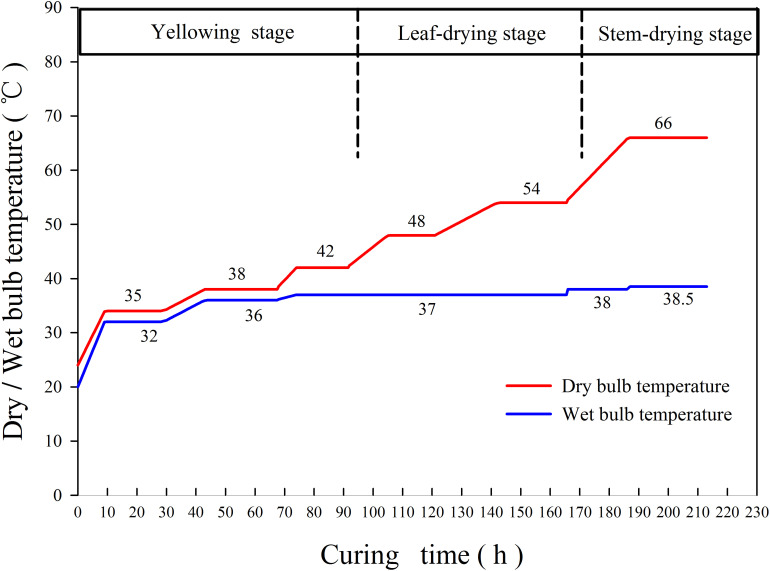
Common curing technology for bulk curing barns in tobacco-growing areas in Laojun Mountain Town, Jianchuan County (variety: Honghuadajinyuan).

The tobacco is packed in three layers, 100–120 leaves per rod and 150–170 rods per layer. The tobacco curing process was mainly carried out according to the local main curing technology (Figure 1). The whole curing process can be divided into three stages: yellowing stage (35–42°C), leaf-drying stage (42–54°C), and stem-drying stage (60–66°C). In the yellowing stage, the original dry bulb and wet bulb temperatures were 24 and 20°C, respectively. The temperature of the dry bulb and that of the wet bulb were raised to 34 and 32°C with temperature rise rates of 1.1°C h^–1^ (dry bulb) and 1.33°C h^–1^ (wet bulb), respectively, and curing for 18 h at a steady temperature. Then, the temperature of the dry bulb and that of the wet bulb were raised to 38 and 36°C at a heating rate of 0.5°C h^–1^, respectively. After curing for 23.5 h at stable temperature, the leaf-drying stage occurred. In the leaf-drying stage, the dry bulb temperature was increased to 42°C at a heating rate of 0.5°C h^–1^, whereas the wet bulb temperature was controlled to 37°C, and drying occurred for 16.5 h at stable temperature. The dry bulb temperature was then increased to 48°C at a heating rate of 0.5°C h^–1^, whereas the wet bulb temperature was unchanged, and curing occurred for 15 h at a stable temperature. Finally, the dry bulb temperature was increased to 54°C at a heating rate of 0.5°C h^–1^, whereas the temperature of the wet bulb remained unchanged. After curing for 22.5 h at stable temperature, transfer to the stem-drying stage occurred. In the stem-drying stage, the dry bulb temperature was increased to 66°C at a heating rate of 1°C h^–1^, whereas the wet bulb temperature was controlled to 38°C, and the curing proceeded for 26 h at stable temperature.

### Data Acquisition and Index Determination

#### Meteorological Data and Sample Collection

The daily meteorological data from August, including temperature, hygrometer, rainfall, and wind speed, were collected by a meteorological station located. The meteorological data collected during the period when local tobacco leaves suffered cold stress were intensively analyzed. All tobacco samples were picked from the middle leaves (5th–10th leaves from bottom to the top) and collected before curing (fresh tobacco leaves), during the critical period of curing process (at the end of 38, 42, 48, and 54°C), and at the end of curing. In each stage, samples comprising 10 tobacco leaves were separately taken for the determination of various indices.

### Structures of Slices of Fresh Tobacco Leaves

The microstructures of fresh tobacco leaves before harvesting and curing were observed by the following method: small pieces of about 0.5 cm × 0.5 cm between the right leaf tip and the sixth to seventh branch veins of the leaf samples were fixed FAA (Formaldehyde, Acetic acid, and Alcohol) fixing solution. A conventional paraffin section method, with slice thickness of 10 μm, was used for hematoxylin staining and sealed with Canadian gum seal to make permanent sections for observation and determination by an Olympus microscope. A total of two sections were observed, and five visual fields were observed and averaged in each section. The observation indices were leaf thickness, upper epidermis thickness, lower epidermis thickness, palisade tissue thickness and sponge tissue thickness, and the data were statistically analyzed.

### Determination of SPAD Value of Tobacco Leaves

A SPAD-502 chlorophyll meter (Konica Minolta Company, Japan) was used to determine the SPAD value of tobacco leaves at each sampling stage; the measuring accuracy was within ±1.0 SPAD unit. The measuring position was from the symmetrical leaf margin and the middle part between the main vein of tobacco leaf, with three parts of the left and right half of the leaf measured, and the average taken as the measure of chlorophyll.

### Determination of the Change of Water Loss Rate of Tobacco Leaves

Fresh tobacco leaves dried with surface moisture, and the samples in each stage of the curing process were weighed by electronic balance (American Shuangjie SA-200Y). The weights were recorded as FW0 and FWn (38, 42, 48, and 54°C), respectively. Then the fresh tobacco leaves and the samples of each stage in the curing process were repeatedly put into a blast drying box and dried at 60°C for 1 h at 105°C. The samples were weighed again, which were recorded as DW0 and DWn (38, 42, 48, and 54°C), respectively.

$$\begin{array}{c} \text{Moisture content of fresh tobacco leaves(\%)}\\ =[(\text{FW}0-\text{DW}0)÷\text{FW}0]\times 100\% \end{array}$$

Moisture content of tobacco leaves at different curing stages (%)

$$\begin{array}{l} \text{= [(FWn}-\text{DWn) }÷\text{ FWn]}\times \text{100\%}\\ \text{ Water loss rate of tobacco leaves (}\%\text{)}\\ \text{ = [(FW0}-\text{FWn) }÷\text{ (FW0}-\text{DW0)]}\times \text{100\%} \end{array}$$

### Determination of Physiochemistry and Chloroplast Pigment Indexes of Tobacco Leaves

Immediately after sampling in each stage, leaves were put into liquid nitrogen and transferred to a freezer at −80°C to determine antioxidant enzymes, including SOD (kit [NBT method], product number: SOD-1-Y); POD (kit, product number: POD-1-Y); CAT (kit [UV absorption method], product number: CAT-1-Y); MDA (kit, product number: MDA-1-Y); PPO (kit, product number: PPO-1-Y). The activity of each enzyme was determined by the kit produced by Suzhou Keming Biotechnology Co., Ltd.

Six main compounds (i.e., starch, total sugar, reducing sugar, total nitrogen, nicotine, and protein) of tobacco samples in different stages were detected using the following methods: total sugar and reducing sugar were analyzed according to Tobacco and Tobacco Products—Determination of Water Soluble Sugars—Continuous Flow Method (YC/T159-2002); total nitrogen was analyzed based on Tobacco and Tobacco Products—Determination of Total Nitrogen—Continuous Flow Method (YC/T161-2002); nicotine was analyzed by utilizing Tobacco and Tobacco Products—Determination of Nicotine—Continuous Flow Method (YC/T160-2002); starches were measured by referring to Tobacco and Tobacco Products–Determination of Starch—Continuous Flow Method (YC/T 216-2014). In terms of polyphenols, the chlorogenic acid, scopoletin, rutin, neochlorogenic acid, caffeic acid, lutein, β-carotene, and total phenols were measured using the method recommended in YC/T 202–2006.

The chloroplast pigment index (chlorophyll and carotenoids) in tobacco leaves at different stages were determined by high-performance liquid chromatography method (YC/T 382-2010). The total chloroplast pigment is the sum of chlorophyll *a*, chlorophyll *b*, and carotenoids.

### Determination of Economic Characters and Sensory Evaluation of Flue-Cured Tobacco Leaves

The flue-cured tobacco leaves were graded according to the national standard GB2635-92, and the tested tobacco leaves were evaluated by the grading hands of the local tobacco station. The prices were all local prices in the same year. Statistics were made for the proportion and average price of high and medium flue-cured tobacco leaves after each treatment.

Sensory quality measurement was evaluated by seven experts from Yunnan Tobacco Technology Center. According to the sensory quality evaluation standard of Yunnan Chinese tobacco, the smoking index can be divided into style characteristics (aroma note), aroma characteristics (aroma volume and aroma quality), smoke characteristics (concentration, mixed gas, strength, and irritancy), and taste characteristics (taste, cleanliness, and moisture) for a total of 100 points.

### Data Statistics

The data were analyzed by the general linear model program, with multiple regression in the SAS 9.3 computer package (SAS Institute Inc., Cary, NC, United States). Salicylic acid level was regarded as a discrete variable and was not analyzed as a quantitative variable because it was not intended to interpolate the effects of salicylic acid over the 0 to 0.1 mol L^–1^ rate range. Values used for statistical analysis represent the mean of three biological replicates. The significance of statistical analysis of all data was based on *P* < 0.05. The data were separated by Tukey (honestly significant difference) test in 95% confidence intervals. Charts were produced by Sigma Plot 12.5 (Systat Software Inc., United States) and Origin 6.0 (Microcal Software, Inc., United States).

## Results

### Changes in Meteorological Factors Generating Field Cold Stress

**FIGURE 2:**
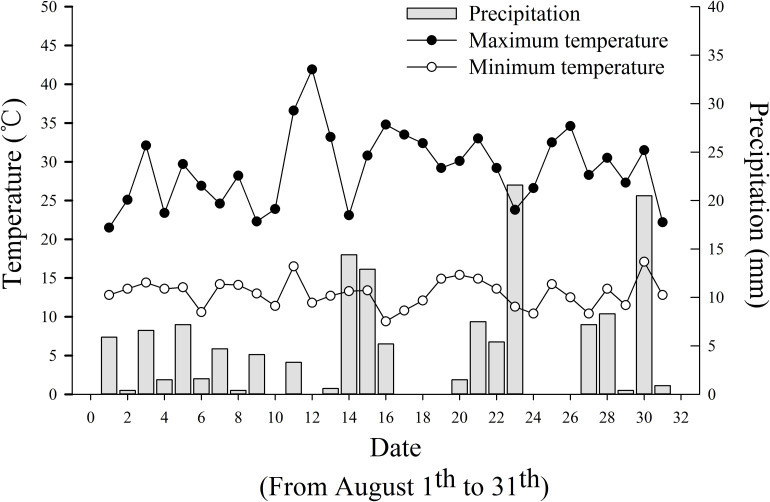
Daily precipitation, the highest and lowest temperatures from August 01 to 31 in Jianji village, Laojun Mountain Town, Jianchuan County.

**FIGURE 3:**
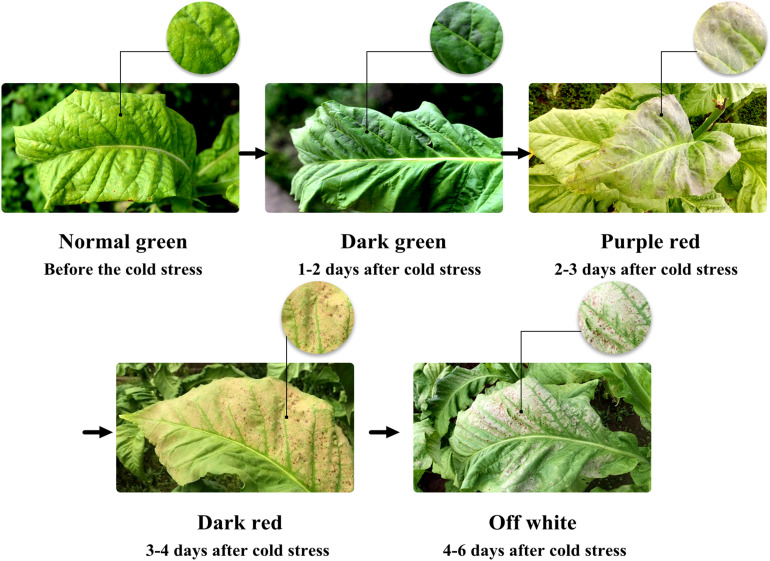
Dynamic symptoms of tobacco after cold stress.

The experiment site located in Jianchuan village of Laojun Mountain Town showed significant fluctuations in temperature and rainfall in August (Figure 2). The daily temperature fluctuated between 21.5 and 41.9°C (high) and 9.4 to 17.1°C (low). The daily precipitation varied within 0–21.6 mm. The maximum diurnal temperature difference (30.1°C) was recorded on August 12; however, no rainfall occurred thereon. The minimum diurnal temperature difference (8.7°C) was found on August 01, accompanying with 5.9 mm of precipitation. Substantial cooling accompanying meteorological disasters (including rainfall and a small amount of hail) appeared on August 16 and 17; in the next 6 days, tobacco showed a range of symptoms caused by cold stress (Figure 3). The tobacco leaves in the middle and upper parts suffered the most serious damage. The surface color of the tobacco leaves changed from normal to dark green (within 1–2 days), to purple red (within 2–3 days), then dark red (within 3–4 days), and finally off-white (within 4–6 days). Eventually, a large area of scalded leaves was found, showing poor flue-curing availability.

### Comparison of Flue-Cured Tobacco Slices Treated With Different Salicylic Acid Concentrations

**FIGURE 4:**
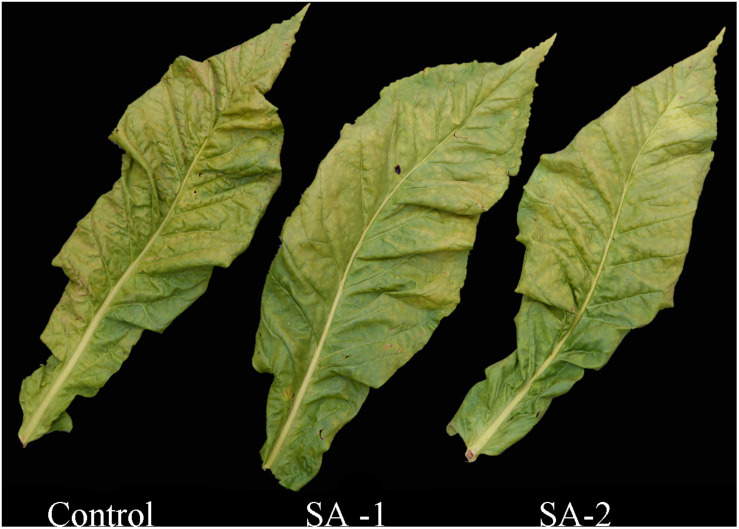
Comparison of leaf appearance in fresh tobacco leaves treated with different salicylic acid concentrations.

In the appearance comparison of fresh tobacco leaves treated with different salicylic acid concentrations, there was no significant difference in the color and leaf size of each treatment, but there was a yellow–gray area on the surface of the Control treated tobacco leaves and no such apparent phenomenon in other treatments (Figure 4).

**FIGURE 5:**
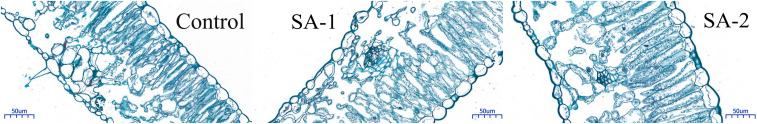
Tissue structure of fresh tobacco leaves treated with different salicylic acid concentration.

There were significant differences in blade thickness, palisade tissue thickness, spongy tissue thickness, and palisade tissue thickness/sponge tissue thickness (tissue ratio) among the treatments (*P* < 0.05) (Figure 5 and Table 1). The palisade tissue thickness, upper epidermal thickness, and lower epidermal thickness treated by SA-2 and SA-1 were significantly higher than those of the Control. The palisade tissue thicknesses in SA-1 treatment were 16 and 178% higher than those treated with SA-2 and Control, respectively. The upper epidermal and lower epidermal thicknesses in SA-2 treatment were 5, 158, and 110%, 129% higher than those treated with SA-1 and Control, respectively. The spongy tissue thickness and blade thickness were SA-1 > SA-2 > Control.

**TABLE 1:** Tissue structure parameters of fresh tobacco leaves treated with different salicylic acid concentration.

Treatment	Thickness of upper epidermis (μm)	Palisade tissue thickness (μm)	Sponge tissue thickness (μm)	Thickness of lower epidermis (μm)	Blade thickness (μm)	Palisade tissue thickness/sponge tissue thickness (μm)
Control	12.54B	62.72B	80.29C	10.75B	166.31C	0.78A
SA-1	30.88A	174.26A	176.72A	22.16A	404.03A	0.99A
SA-2	32.32A	149.8A	141.46B	24.59A	348.17B	1.06A

*Different capital letters indicate significant differences between treatments at the same stage (P < 0.05) (n = 3).*

### Comparison of Water Loss Rate of Flue-Cured Tobacco Leaves in Treatment With Different Salicylic Acid Concentrations

**FIGURE 6:**
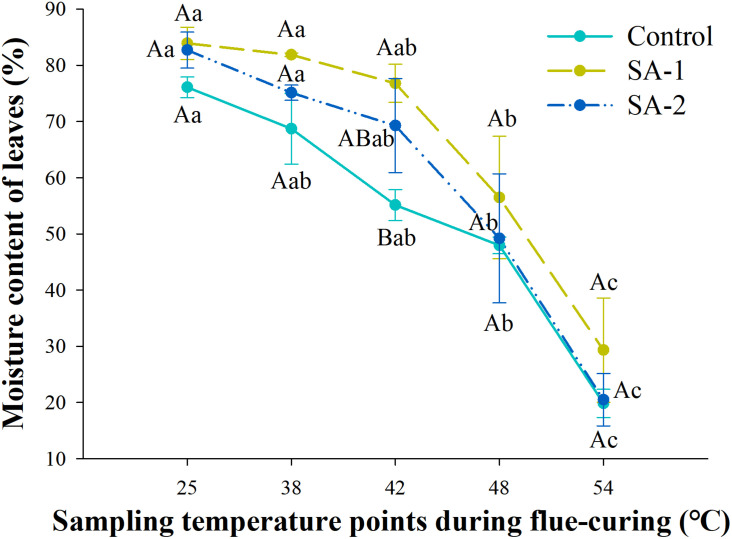
Change of moisture content during bulk curing of flue-cured tobacco with different salicylic acid concentrations. Different capital letters indicate significant differences between treatments at the same stage (*P* < 0.05). Different lowercase letters indicate significant differences between the same treatments at different curing stages. Data are presented as means ± standard error (*n* = 3).

**FIGURE 7:**
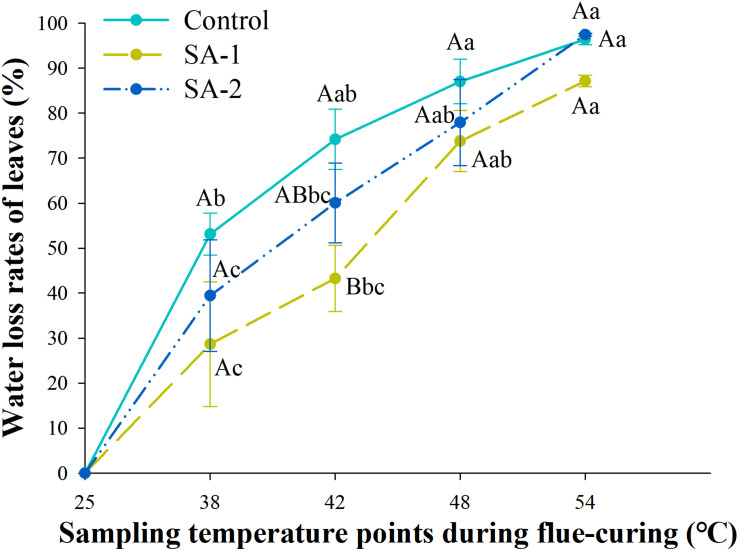
Change in water loss rate during bulk curing of flue-cured tobacco with different salicylic acid concentrations. Different capital letters indicate significant differences between treatments at the same stage (*P* < 0.05). Different lowercase letters indicate significant differences between the same treatments at different curing stages. Values represent the averages of three biological replicates. Data are presented as means ± standard error (*n* = 3).

With the advance of curing time, the moisture content in all treatments declined. There were significant differences (*P* < 0.05) in water loss and moisture content of tobacco leaves in different curing stages (Figures 6, 7). There are no significant differences in water loss and moisture content of tobacco leaves in the same curing temperature.

Under the water content index, the change trend was significant between SA-1 and Control and SA-2 at 25–48°C. At 25–48°C, SA-1 was 10–39% higher than Control, whereas SA-2 was 3–26% higher than Control. The SA-1 and SA-2 treatments were significantly higher than Control. The moisture content of SA-1 and SA-2 treatments quickly decreased after 42°C, the moisture content decreased 62 and 70%, respectively, at 54°C compared with 42°C (Figure 6). Under the index of water loss rate, at 38–54°C, the change trend was for a significant difference between Control and SA-1 and SA-2; Control increased by 11–85% and 12–35% compared with SA-1 and SA-2, respectively, and SA-2 increased by 6–39% compared with SA-1. The SA-1 treatments were significantly higher than SA-2 and Control at 42°C. Compared with 38°C, the water loss rate of Control, SA-1, and SA-2 increased by 81, 204, and 147%, respectively (Figure 7).

### Comparison of SPAD Values and Chloroplast Pigments in Tobacco Leaves With Different Salicylic Acid Concentrations During Flue-Curing Process

There were significant differences (*P* < 0.05) in SPAD, chlorophyll *a*, and chlorophyll *b*, among three concentrations of salicylic acid in flue-cured tobacco leaves (Table 2).

**TABLE 2:** Changes of SPAD and chloroplast pigments in different curing stages with different salicylic acid concentrations.

Treatment	Sampling temperature (°C)	Continuous curing time (h)	SPAD	Chlorophyll *a* (μg/g)	Chlorophyll *b* (μg/g)	Lutein (μg/g)	β-Carotene (μg/g)
Control	Fresh tobacco leaves	–	35.6Aa	154.04Ba	78.27Ba	137.73Aa	2,089.93Aa
	38	23.5	8.7Abc	24.9Ab	13.21Ab	129.38Aa	2,046.98Ba
	42	16.5	5.8Ac	16.41Bb	10.54Ab	142.05Ba	2,170.23Ba
	48	15	6.2Ac	9.81Ab	4.45Ab	127.61Aa	2,351.72Aa
	54	22.5	12.9Ab	11.5Ab	7.06Ab	128.42Aa	2,173.94Aa
	Initially flue-cured tobacco leaves	–		11.32Ab	7.77Ab	136.5Aa	2,350.62Ba
SA-1	Fresh tobacco leaves	–	40.8Aa	238.05Aa	142.1Aa	170.07Ab	2,116.48Ac
	38	23.5	6.6Ab	28.79Abc	16.4Ab	186.32Ab	2,357.03ABbc
	42	16.5	5.5Ab	111.49Ab	56.93Ab	291.5Aa	4,048.59Aa
	48	15	5.9Ab	7.46Ac	4.32Ab	146.93Ab	2,648.99Abc
	54	22.5	4.5Bb	3.27Ac	2.83Ab	197.09Ab	3,077.14Aabc
	Initially flue-cured tobacco leaves	–		12.08Ac	7.45Ab	199.85Ab	3,386.15ABab
SA-2	Fresh tobacco leaves	–	40.2Aa	232.35ABa	161.56Aa	196.78Aa	2,544.12Aab
	38	23.5	10.9Ab	57.55Ab	22.84Ab	197.26Aa	3,265.91Aab
	42	16.5	5.9Ab	12.28Bb	6.36Ab	142.36Ba	2,664.09Bab
	48	15	10.8Ab	6.35Ab	2.88Ab	141.25Aa	2,531.51Aab
	54	22.5	8.8ABb	4.85Ab	4.14Ab	149.45Aa	2,193.89Ab
	Initially flue-cured tobacco leaves	–		8.8Ab	5.63Ab	209.69Aa	3,559.81Aa

*Different capital letters indicate significant differences between treatments at the same stage (P < 0.05). Different lowercase letters indicate significant differences between the same treatments at different curing stages. Values represent the averages of three biological replicates.*

During the curing process, the SPAD, chlorophyll *a*, and chlorophyll *b* contents of each treatment declined, and degradation the fastest at 38°C.

For the SPAD index, the content of Control was the lowest in the fresh tobacco stage. For the chlorophyll *a* and *b* indices, SA-1 was higher than Control when the curing stage was fresh tobacco to 42°C.

For the SPAD index, SA-1 and SA-2 were 15% and 13% higher than Control in fresh tobacco leaves, respectively. For the chlorophyll *a* and chlorophyll *b* index, SA-1 was 16–579% and 24–440% higher than Control in the curing stage of fresh tobacco leaves to 42°C, and the difference was not significant in the later stage. For the lutein index, SA-1 and SA-2 were 15–105% and 0.22–54% higher than Control. For the β-carotene index, SA-1 and SA-2 were 1–87% and 1–60% higher than Control.

### Comparison of Chemical Components and Polyphenols Contents in Flue-Cured Tobacco Leaves Treated With Different Salicylic Acid Concentrations

The ratios of starch, total sugar, reducing sugar, and sugar alkali in the chemical composition indices of tobacco leaves treated with salicylic acid were significantly different from that of Control (Tables 3, 4). There are significant differences in the ratios of chlorogenic acid and rutin in the polyphenols.

**TABLE 3:** Contents of chemical compounds during bulk curing of flue-cured tobacco with different salicylic acid concentrations.

Treatment	Sampling temperature (°C)	Curing time (h)	Total sugar (%)	Reducing sugar (%)	Total nitrogen (%)	Nicotine (%)	Potassium oxide (%)	Water soluble chlorine (%)	Starch (%)	Protein (%)	Ratio of sugar to alkali (%)	Ratio of nitrogen to alkali (%)
Control	Fresh tobacco	–	6.32Ab	4.55Ab	1.69Aa	2.26ABb	1.58Aa	0.24Ab	41.36Aa	8ABa	3.01Ac	0.77Aa
	38	23.5	37.04Aa	24.53Aa	1.74Ba	1.84Bb	1.68Aa	0.49Aab	8.86Abc	5.83Bb	22.48Aa	1.16Aa
	42	16.5	36.45Aa	25.85Aa	1.69Ba	2.14Bb	1.42Ca	0.29Bb	9.58Ab	6.17Ab	18.44Aab	0.83Aa
	48	15	34.06Aa	22.6Aa	2.04Aa	3.23Aa	1.77Aa	0.41Aab	5.19Ac	6.49Ab	10.58Abc	0.64Aa
	54	22.5	34.77Aa	20.99Aa	1.75Aa	2.2Ab	1.92Aa	0.62Aa	7.81Abc	6.11Ab	15.95Aab	0.8Aa
	Primary tobacco leaf	–	33.61Aa	23.52Aa	1.94ABa	2.59Aab	1.7Ba	0.46Aab	7.95Abc	6.44Bb	13.04Aab	0.76Aa
SA-1	Fresh tobacco	–	7.62Ac	4.98Ac	1.62Ab	1.66Bb	1.45Ac	0.31Ab	41.91Aa	6.9Ba	4.89Aa	0.98Aa
	38	23.5	32.07ABa	19.75ABa	1.94Bab	2.85Aa	1.8Abc	0.56Aab	8.61ABb	6.13Ba	11.37Ba	0.68Ba
	42	16.5	20.75Bb	12.9Bb	2.2Aa	3.1Aa	3.06Aa	0.87Aa	3.07Bcd	7.04Aa	7.08Ba	0.71Aa
	48	15	35.25Aa	21.95Aa	1.87Aab	3Aa	1.58Abc	0.55Aab	4.02Acd	5.88Aa	11.85Aa	0.63Aa
	54	22.5	32.62Aa	18.41Aab	2.01Aab	2.84Aa	2.16Ab	0.52Aab	1.79Bd	5.79Aa	12.05Aa	0.72Aa
	Primary tobacco leaf	–	31.72ABa	22.01ABa	1.92Bab	2.4Aab	2.12ABb	0.59Aab	6.97Abc	6.64Ba	13.21Aa	0.8Aa
SA-2	Fresh tobacco	–	4.39Ac	2.42Ab	1.98Aab	2.65Ab	1.43Ab	0.36Aa	35.94Ba	8.9Aa	1.64Ab	0.75Aa
	38	23.5	28.03Bab	16.79Ba	2.59Aa	3.59Aa	1.95Aab	0.68Aa	4.32Bb	7.9Aabc	7.92Bab	0.72Ba
	42	16.5	27.63Bab	19.48Aa	2.31Aab	3.18Aab	2.07Bab	0.54ABa	5.03Bb	7.28Aabc	8.83Bab	0.73Aa
	48	15	34.19Aa	20.49Aa	1.93Ab	2.96Aab	1.81Aab	0.53Aa	3.68Ab	6.11Ac	11.71Aa	0.65Aa
	54	22.5	33.13Aa	21.35Aa	1.96Aab	3.08Aab	1.91Aab	0.48Aa	3.62ABb	6.72Abc	12.91Aa	0.67Aa
	Primary tobacco leaf	–	25.35Bb	16.82Ba	2.35Aab	3.08Aab	2.34Aa	0.68Aa	4.41Ab	8.28Aab	8.28Aab	0.76Aa

*Different capital letters indicate significant differences between treatments at the same stage (P < 0.05). Different lowercase letters indicate significant differences between the same treatments at different curing stages. Values represent the averages of three biological replicates.*

**TABLE 4:** Contents of polyphenols during bulk curing of flue-cured tobacco with different salicylic acid concentrations.

Treatment	Sampling temperature (°C)	Curing time (h)	Neochlorogenic acid (mg/g)	Chlorogenic acid (mg/g)	Caffeic acid (mg/g)	Scopoletin (mg/g)	Rutin (mg/g)	Kaempferol-3-*O*-rutinoside (mg/g)
Control	Fresh tobacco	–	0.82Ad	8.78Ab	0.13ABc	0.23Aa	7.39Ab	0.08Bb
	38	23.5	1.85Ac	16.59Ba	0.17Abc	0.08Ab	11.35Ba	0.17Aa
	42	16.5	2.08Bbc	13.68Aab	0.18Abc	0.07Ab	12.16Ba	0.08ABb
	48	15	2.95Aa	18.43Aa	0.28Aa	0.11Ab	12.88Aa	0.09Ab
	54	22.5	2.54Aabc	17.61Aa	0.28Aa	0.06ABb	12.71Aa	0.08Ab
	Primary tobacco leaf	–	2.69Aab	18.22Aa	0.25Aab	0.1Ab	12.25Aa	0.06Ab
SA-1	Fresh tobacco	–	1.02Ab	10.05Ac	0.18Ac	0.15Ba	7.64Ac	0.13Ab
	38	23.5	2.46Aa	18.46ABab	0.17Ac	0.09Aab	13.17ABab	0.19Aa
	42	16.5	2.99Aa	10.91Ac	0.17Ac	0.09Aab	10.32Bbc	0.12Abc
	48	15	2.88Aa	21.72Aa	0.28Aa	0.12Aab	14.16Aa	0.08Ac
	54	22.5	3.01Aa	13.7Abc	0.19Bbc	0.12Aab	10.12Abc	0.1Abc
	Primary tobacco leaf	–	2.83Aa	17.25Aab	0.27Aab	0.06Ab	11.97Aab	0.08Ac
SA-2	Fresh tobacco	–	0.68Ac	5.53Ac	0.08Bb	0.18ABa	6.82Ab	0.09ABb
	38	23.5	2.28Ab	22.06Aa	0.22Aa	0.12Aabc	15.42Aa	0.19Aa
	42	16.5	2.95Aab	15.74Ab	0.2Aa	0.14Aab	16.34Aa	0.07Bb
	48	15	2.93Aab	19.38Aab	0.23Aa	0.09Abc	15.29Aa	0.08Ab
	54	22.5	3Aab	17.24Aab	0.27ABa	0.04Bc	13.3Aa	0.06Ab
	Primary tobacco leaf	–	3.11Aa	17.97Aab	0.22Aa	0.1Abc	13.68Aa	0.08Ab

*Different capital letters indicate significant between different treatments at the same stage (P < 0.05). Different lowercase letters indicate significant between the same treatments at different curing stages. Values represent the averages of three biological replicates.*

With the advance of curing time, the starch content of the treated tobacco leaves decreased; the fastest decline was at 38°C. The ratio of total sugar, reducing sugar, and sugar alkali increased rapidly at first and then decreased slowly, and increased rapidly at 38°C stage of the fresh tobacco leaves.

Under different curing stages, the change trend of carbon metabolite index was Control > SA-1 > SA-2. The total sugar content of Control was 6–76% and 5–44% higher than that of SA-1 and SA-2; the reducing sugar content of Control was 3–100% and 10–88% higher than that of SA-1 and SA-2, and the starch content of Control was 3–336% and 15–116% higher than that of SA-1 and SA-2, respectively. Under different curing stages, the change trend of nitrogen metabolite index was as follows: SA-2 > SA-1 > Control. The total nitrogen content of SA-2 was 12–49% and 3–34% higher than Control and SA-1, the nicotine content of SA-2 was 17–95% and 3–60% higher than CK and SA-1, and the protein content of SA-2 was 10–36% and 3–29% higher than Control and SA-1, respectively.

The chlorogenic acid and rutin of each treatment increased rapidly from fresh tobacco leaves to 38°C and then stabilized gradually. Under the chlorogenic acid index, the 38°C of Control, SA-1, and SA-2 treatment increased 89, 84, and 299%, respectively, compared with the fresh tobacco leaf stage; under the rutin index, the 38°C of Control, SA-1, and SA-2 treatment increased 54, 72, and 126%, respectively, compared with the fresh tobacco leaf stage.

### Comparison of Activities of PPO and Antioxidant Enzymes in Tobacco Leaves With Different Salicylic Acid Concentrations During Flue-Curing

**FIGURE 8:**
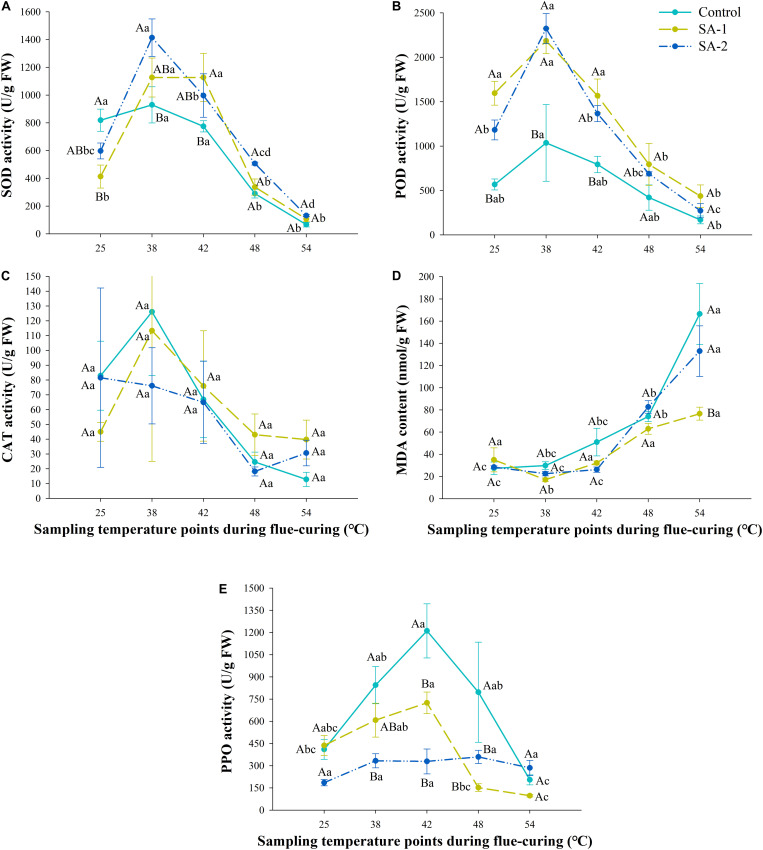
Antioxidant enzyme system and polyphenol oxidase activity during bulk curing of flue-cured tobacco with different salicylic acid concentrations. **(A)** Effect of treatment with different salicylic acid concentrations on SOD activity at different curing stages. **(B)** Effect of treatment with different salicylic acid concentrations on POD activity at different curing stages. **(C)** Effect of treatment with different salicylic acid concentrations on CAT activity at different curing stages. **(D)** Effect of treatment with different salicylic acid concentrations on the content of MDA at different curing stages. **(E)** Effect of treatment with different salicylic acid concentrations PPO activity at different curing stages. Different capital letters indicate significant differences between treatments at the same stage (*P* < 0.05). Different lowercase letters indicate significant differences between the same treatments at different curing stages. Data are presented as means ± standard error (*n* = 3).

For the SOD and POD activity indices, there is a rule of SA-2 > SA-1 > Control, and under the CAT index, there is a rule of Control > SA-1 > SA-2 (Figure 8). Except for the SA-2 treatment under the CAT index, the three enzymes show a change trend of increasing first and then decreasing. The three enzymes have the highest activity at the curing stage of 25–42°C and then a sharp decline. The MDA content of different treatments increased with the curing process, showing the rule of Control > SA-1 > SA-2. The growth rate and content of Control treatment at 25°C were higher than SA-1 and SA-2; the growth rate and content of SA-2 treatment at 42°C were higher than SA-1. The PPO activity of each treatment was Control > SA-1 > SA-2. The PPO activity of Control and SA-1 increased first and then decreased sharply, reaching the peak at 42°C; the PPO activity of SA-1 at 38–48°C was higher than that at 25 and 54°C.

### Comparison of Economic Traits and Sensory Evaluation of Flue-Cured Tobacco Leaves Within Different Salicylic Acid Concentrations

**FIGURE 9:**
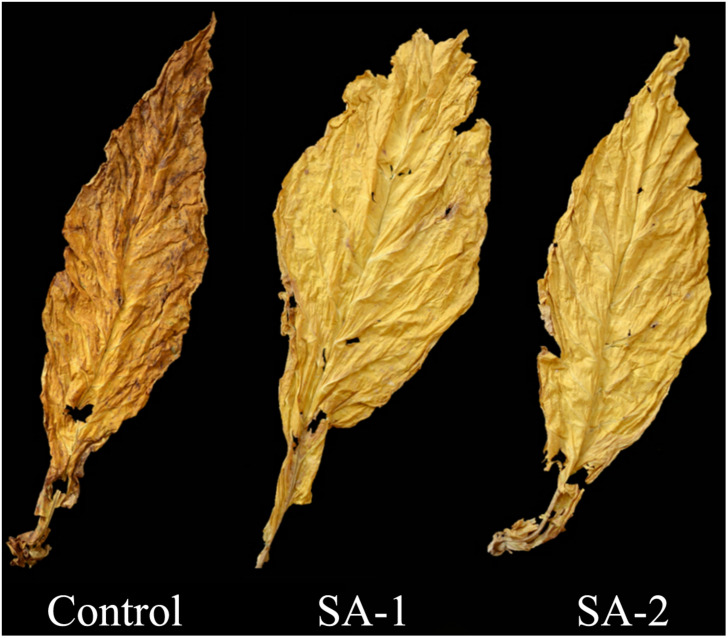
Comparison of appearance of flue-cured tobacco after curing with different salicylic acid concentrations.

There were significant difference in appearance between CK and SA-1 and SA-2 treated primary flue-cured tobacco leaves; SA-1 and SA-2 treated tobacco leaves were bright in color, without obvious variegation and browning; CK treated tobacco leaves were dark in color, with small leaf opening, and obvious visible variegation and browning on the surface (Figure 9).

There were significant differences between Control and SA-1 and SA-2 in the yield, output value, proportion of middle and top grade tobacco, average price, and sensory evaluation quality of primary flue-cured tobacco leaves (Tables 5, 6); Control treatment was worst. The yield, output value, proportion of middle and top grade tobacco, and average price of SA-1 was 21, 63, 53, and 35% higher than Control, and SA-2 was 25, 51, 25, and 22% higher than Control, respectively. In sensory quality, the total score of SA-2 treatment was higher than that of SA-1 treatment by 4%, significantly higher than Control treatment by 22%; SA-1 treatment was higher than Control treatment by 17%.

**TABLE 5:** Economic properties of flue-cured tobacco with different salicylic acid concentrations.

Treatment	Yield (kg ⋅ ha^–1^)	Output value (dollar ⋅ ha^–1^)	Proportion of middle and top grade tobacco (%)	Average price (dollar ⋅ kg^–1^)
Control	1,979B	5,778B	57B	2.92B
SA-1	2,392A	9,407A	87A	3.93A
SA-2	2,464A	8,738A	71AB	3.55AB

*Different capital letters indicate significant differences between treatments at the same stage (P < 0.05). Values represent the averages of three biological replicates.*

**TABLE 6:** Sensory evaluation of smoking quality of flue-cured tobacco with different salicylic acid concentrations.

Treatment	Aroma note (10)	Aroma quality (15)	Aroma volume (15)	Concentration (10)	Mixed gas (10)	Irritancy (15)	Strength (5)	Cleanliness (10)	Moisture (5)	Taste (5)	Total score (100)
Control	6	12	11.5	6.5	6.5	13.5	3.5	6	3	3.5	72B
SA-1	8.5	14	13	8	6.5	13	4	8.5	4.5	4	84A
SA-2	8.5	13.5	13.5	8.5	7.5	14.5	4.5	8.5	4	4.5	87.5A

*Different capital letters indicate significant differences between treatments at the same stage (P < 0.05). Values represent the averages of three biological replicates.*

## Discussion

### Effects of Salicylic Acid on Quality and Curing Characteristics of Fresh Flue-Cured Tobacco Leaves Under Cold Stress

Spraying salicylic acid can effectively prevent or alleviate the stress effect of cold injury on the tissue structure, photosynthetic system, and yellowing of flue-cured tobacco leaves during the harvesting and curing period (Dat et al., 2001; Zhu et al., 2013). Spraying salicylic acid significantly increased the thickness of palisade tissue, upper epidermal and lower epidermal, compared with the Control. This result could be related to the ability of leaf secretion; SA is shown to alleviate and regulate various cold-induced changes (Saleem et al., 2020). After cold stress, plant leaves will significantly thicken because of the secretion of a metabolic wax layer and a large amount of lignin, and palisade tissue and spongy tissue will significantly shrink to cope with water loss and wilting (Li et al., 2004, 2011). A suitable concentration of salicylic acid could increase the activity of antioxidant enzymes such as SOD and POD in leaves, maintain the stability of membrane system, reduce the loss of leaf tissue cells, alleviate the damage caused by cold damage to leaf tissue structure, and thus improve the cold resistance of flue-cured tobacco in the period of harvesting and curing (Janda et al., 1999; Kang and Saltveit, 2002; Khan et al., 2015). This study showed that the quality of tobacco leaves with high concentration of salicylic acid was not as good as that with low concentration. Existing studies also show that with increasing salicylic acid concentration, the membrane permeability increases rapidly, and the high concentration of salicylic acid increases the damage of low temperature on plants (Wang et al., 2009). Meanwhile, SA plays an important role in improved photosynthetic system and chloroplast pigment content in stressed plants (Fariduddin et al., 2003). In our study, chloroplast pigment content was SA-1, SA-2 > Control in fresh tobacco leaves. This result could be related to membrane system. The chloroplast membrane is protected by spraying salicylic acid and thus increases the content of chloroplast pigment (Huang et al., 2016). The plastid pigment content with a low concentration of salicylic acid spraying was significantly higher than that of high concentration and no spraying of salicylic acid, which shows that low-concentration salicylic acid spraying can effectively guarantee the vigorous light cooperation of tobacco plant use.

The coordination of water loss and yellowing is an important link to determine the quality of tobacco leaves. The fastest water loss rate was at 42–48°C, and the water loss rate of each treatment per unit time was Control > SA-2 > SA-1. The degradation rate of chloroplast pigment in each treatment was Control > SA-2 > SA-1, which indicated that the rate of water loss and yellowing of tobacco leaves without salicylic acid treatment was too fast in the process of tobacco curing, and the time provided for the transformation of contents and the formation of tobacco quality in the yellowing and fixation period was far from enough. This result could be related to the ability of cuticular wax layer. Exogenous salicylic acid can induce the increase in the total amount and component content of cuticular wax in plant leaves, preventing damage of low temperature to the internal structures of plants, and effectively reduce the water loss through transpiration and stomata of cuticle, thus reducing plant water loss rate (Kosma et al., 2009; Ni et al., 2013; Zhu et al., 2017; Wu et al., 2018). And when salicylic acid is not sprayed, the tissue cells of tobacco leaves were ruptured due to the damage of plasma membrane, the water loss was too fast, and it is easy to be browned by enzyme and cause browning tobacco in a large area (Ghanbari and Sayyari, 2018). Therefore, compared with the treatment without spraying, spraying salicylic acid can effectively ensure that the yellowing and water loss can be carried out simultaneously, and the effect is better at a lower concentration, which can ensure the orderly transformation of the internal substances and greatly alleviate the curing difficulty caused by cold injury stress.

### Effect of Salicylic Acid on Physiological and Biochemical Characteristics of Cold-Stressed Flue-Cured Tobacco

By spraying appropriate concentration of salicylic acid, the activity of the antioxidant enzyme system and the accumulation of MDA in flue-cured tobacco can be increased (Cai et al., 2003). Meanwhile, these results showed that the low salicylic acid treatment could ensure the cell integrity of tobacco leaves, maintain water loss and yellowing in the curing process in an orderly manner, and reduce browning tobacco and invertase deactivation caused by cell damage and rapid water loss during the yellowing and fixation period. When flue-cured tobacco receives a low temperature signal, SOD activity in tobacco leaves increases rapidly. It decomposes the O_2_^–^ and H_2_O_2_ produced by superoxide in the plant under cold stress together with CAT and POD. With increasing cold stress, the activity of antioxidant enzymes is inhibited, unable to complete the decomposition function until the oxidation products accumulated in the cell poison the cell membrane and cause damage to plants (Jing et al., 2008). The MDA content reflects the degree of oxidation in plants, and its level is one of the indicators of the degree of stress damage to plants. It can also be used as a reference indicator of the strength of stress tolerance of plants (Liu et al., 2018). In the current study, salicylic acid treatment of flue-cured tobacco seedlings in cold stress can significantly improve the ability to scavenge reactive oxygen species, protect the integrity of cell membrane, and stabilize physiological and biochemical reactions by increasing the activities of POD and SOD, which is consistent with the results of Zhu et al. (2013).

Cold stress can promote the rapid accumulation of polyphenols in tobacco leaves, destroy the integrity of cell membrane, and lead to the combination of PPO in vacuole and polyphenols (Gong, 2011; Ghanbari and Sayyari, 2018). Spraying salicylic acid can effectively alleviate the accumulation of polyphenols, reduce the activity of PPO, and protect the integrity of cell membrane (Han et al., 2017). The PPO activity of the fresh tobacco leaves increased and then decreased in the curing process. The peak value appeared at 42°C, and the PPO activity of each stage was consistent with that of the fresh tobacco leaves. Treatment by low concentrations of salicylic acid could alleviate cold stress in the field, better ensure the orderly enzymatic browning reaction in the tobacco curing process, and reduce the formation of browning tobacco.

### The Effect of Salicylic Acid on Yield and Quality of Cold-Stressed Flue-Cured Tobacco

Salicylic acid had a positive effect on the inhibition of carbon and nitrogen metabolism and aroma matter accumulation of flue-cured tobacco (Dechuan, 2006). Increasing spraying concentration of salicylic acid decreased carbon metabolism and increased nitrogen metabolism. Chang et al. (2012) show that the soluble sugar content in the leaves of soybean seedlings under cold stress will increase after being treated with salicylic acid, and the activity of various enzymes and the capacity for osmotic regulation will increase. This differs from the results of the current study, which may be because the proteins involved in carbohydrate catabolism were also induced by salicylic acid (Cai et al., 2014). Studies have also shown that lower concentrations of salicylic acid can increase the content of carbon metabolites and stress resistance of plants, which is similar to the results of this study (Zhang, 2006; Cui, 2007; Khan et al., 2015).

In this study, the economic characteristics and sensory evaluation quality of the primary flue-cured tobacco leaves treated with low concentrations of salicylic acid were higher than those of the leaves non-treated and treated with high concentration of salicylic acid. Economic loss could be effectively reduced by spraying low concentration of salicylic acid. Low temperature can promote the accumulation of phenolics in plants, inhibit oxygen free radicals, and protect the integrity of light system and cell membrane, so cold stress can promote the accumulation of polyphenols. Because of the destruction of the cell membrane and the strong peroxidation of membrane lipid, PPO activity was strong under conditions of high temperature and high humidity in the curing process (Farooq et al., 2008). After the cell membrane breaks, it easily reacts with a large amount of accumulated polyphenols forming a large area of browning tobacco, reducing the quality of tobacco (Rivera-Pastrana et al., 2010; Liu et al., 2017). By paying attention to weather forecast and long-term accumulated experience of field climate change in high-altitude tobacco planting areas of Yunnan Province, low-concentration salicylic acid can be sprayed before field cold stress, so as to effectively prevent cold stress and ensure high tobacco quality.

## Data Availability Statement

The raw data supporting the conclusions of this article will be made available by the authors, without undue reservation, to any qualified researcher.

## Ethics Statement

Written informed consent for participation in this study was obtained from the participants.

## Author Contributions

CZ and YZ designed the experiments and managed the projects. JC, AX, and GZ performed the experiments. YJ, BH, and XH performed the data analysis. XH, TL, and KR wrote the manuscript. All authors contributed to the article and approved the submitted version.

## Conflict of Interest

YZ was employed by the company Tobacco Monopoly Administration of Yunnan Wenshan Prefecture. The remaining authors declare that the research was conducted in the absence of any commercial or financial relationships that could be construed as a potential conflict of interest.

## Supplementary Material

Supplementary Figure 1 The variation of average temperature and diurnal range in Jianchuan from April 13th to August 31st in 2012 to 2016.
